# Infiltration of M2a macrophages is predominant in genital verruciform xanthoma

**DOI:** 10.1111/1346-8138.17654

**Published:** 2025-02-06

**Authors:** Akira Miyazaki, Tomoki Taki, Shoichiro Mori, Motohito Yamada, Masashi Akiyama

**Affiliations:** ^1^ Department of Dermatology Nagoya University Graduate School of Medicine Nagoya Japan; ^2^ Department of Dermatology Toyohashi Municipal Hospital Toyohashi Japan

**Keywords:** CCR2, CD163, CD68, DC‐SIGN, foam cells

Verruciform xanthoma (VX) is a rare erythematous benign warty tumor that was first reported by Sachs in 1903 and named by Shafer in 1971.^1^ It mainly develops in the genital area and the oral mucosa of the elderly. Histopathologically, papillomatous changes in the epidermis and foam cell infiltration in the dermis are seen. To elucidate the etiology of VX, we immunohistochemically investigated the subtypes of infiltrating macrophages. This study was approved by the ethics committees of Nagoya University Hospital and Toyohashi Municipal Hospital (approval numbers: 2022‐0422 and 736, respectively) and was carried out under the Declaration of Helsinki.

A 71‐year‐old Japanese man (case 1) had a 1.5 × 1‐cm erythematous pedunculated verrucous nodule on the scrotum (Supporting Information Figure S1a,b). The tumor had enlarged slowly in the previous 10 years. A comorbidity was prostate cancer, which was under observation. Hematoxylin–eosin staining of the resected nodule showed abundant foamy macrophages in the dermis (Supporting Information Figure S1c,d). These stained strongly positive for CD80 (an M1 macrophage marker), CD163 (an M2a/M2c macrophage marker), and DC‐SIGN (an M2a macrophage marker) and weakly positive for CD86 (an M1/M2b macrophage marker), but negative for CCR2 (an M2c macrophage marker) (Supporting Information Figure S2a–f). Double‐color immunofluorescence staining revealed a large proportion of the DC‐SIGN‐positive cells to be positive also for CD163 (a macrophage marker) (Supporting Information Figure S3a), suggesting that the DC‐SIGN‐positive cells were mainly macrophages, particularly M2a macrophages. CD80‐ and CD163‐positive cells existed independently (Supporting Information Figure S3b). The type 2‐related molecules IL‐4/13 and TSLP were expressed in the epidermis and around the macrophages (Supporting Information Figure S3c–e). Periostin was more strongly expressed in the dermis in the patients than in the healthy control (Supporting Information Figures S3f, S4, and S5). Periostin can promote M2 macrophage skewing in VX lesions. An 89‐year‐old Japanese man (case 2) had a verrucous papule of 7 mm in diameter on the scrotum. He also had well‐controlled psoriasis vulgaris and had been receiving guselkumab therapy (Supporting Information Figures S6–S8). A 73‐year‐old Japanese man (case 3) had an erythematous pedunculated verrucous nodule of 2 cm in diameter on the scrotum, which enlarged over the course of 10 months (Supporting Information Figures S9–S11). The results of immunohistochemical staining and immunofluorescent double‐staining for infiltrating macrophages were similar to those of case 1, although the staining of DC‐SIGN was weak in case 3. Unlike in the VX lesions, infiltrating macrophages in the xanthelasma palpebrarum lesion were negative for CD80 and CCR2 (Supporting Information Figures S12 and S13).

Clinical features and cell surface markers of infiltrating macrophages in the present three VX cases are summarized in Table 1. These results indicate that M2a macrophage‐predominant infiltration accompanies M1 macrophages in genital VX lesions. Previous studies also reported the infiltration of CD163‐positive M2a macrophages in VX lesions.^2^, ^3^

**TABLE 1:** Clinical features and cell surface markers of infiltrating macrophages in the present three VX cases.

	Age (years)	Sex	Tumor site	Tumor diameter	Cell surface marker of infiltrating macrophages					
					CD68 (pan‐macrophage marker)	CD80 (M1 macrophage marker)	CD86 (M1/M2b macrophage marker)	CD163 (M2a/M2c macrophage marker)	DC‐SIGN (M2a macrophage marker)	CCR2 (M2c macrophage marker)
Case 1	71	Male	Scrotum	1.5 × 1 cm	+++	+	+	+++	++	−
Case 2	89	Male	Scrotum	0.7 cm	+++	+	+	++	++	−
Case 3	73	Male	Scrotum	2 cm	+++	++	+	+++	+	−
Control, xanthelasma paplebrarum	49	Female	Eyelid	−	+++	−	+	+++	+	−

Abbreviation: VX, Verruciform xanthoma.

*Note*: Negative (−), positive (+), strongly positive (++), extremely positive (+++).

M1 macrophages are induced by cytokines, such as interleukin (IL)‐1, IL‐12, IL‐17, and tumor necrosis factor‐α, causing various inflammatory reactions.^4^ M2 macrophages are induced by IL‐4 and IL‐13 and have anti‐inflammatory activity. M2 macrophages consist of three subpopulations: M2a, for tissue remodeling; M2b, for immune regulation and tumor progression; and M2c, for apoptotic cell removal. DC‐SIGN is an M2a‐specific cell surface marker. It is a pattern recognition receptor (PRR) for mannan and galactomannan in pathogen‐associated molecular patterns. It recognizes various kinds of microorganisms, including viruses, bacteria, and fungi.

Due to its predilection sites and verrucous appearance, an association with human papilloma virus (HPV) has been suspected. However, numerous experiments failed to demonstrate HPV,^5^ and HPV infection is not considered to be the major cause of VX. Some have hypothesized that local irritation leads to phagocytosis by macrophages of lipids released from damaged keratinocytes. Although we did not conduct microbial tests of the lesions, the genital region, a common site of VX, is home to various skin resident flora, such as *Candida albicans*. Small but significant numbers of M1 macrophages exist in VX lesions, but such macrophages were not seen in the xanthelasma palpebrarum case (a control subject in the present study). Certain pathological microorganisms or mechanical stimuli in the genital area may contribute to M1 macrophage infiltration. In addition, some VX lesions develop at post‐surgical sites,^6^ post‐traumatic scars, and chronic inflammatory sites,^7^ where dysbiosis tends to occur. Microbial stimulation via PRR activation induces the infiltration of M2a macrophages. The scrotum sometimes shows bleeding as an easily rubbed site, and CD163 works as a hemoglobin scavenger receptor. Hemoglobin or red blood cells might be involved in the pathogenesis, in addition to microbial stimuli. Those microbial and physical stimuli may contribute to VX etiology.

## FUNDING INFORMATION

This work was supported by JSPS KAKENHI under grant numbers JP20K17315, JP21H02941, and JP23K15282, and by the JST SPRING program under grant JPMJSP2125. This work was also supported by a grant from the Ministry of Health, Labor and Welfare of Japan (Health and Labor Sciences Research Grant for Research on Intractable Diseases: 23FC1039).

## CONFLICT OF INTEREST STATEMENT

Prof. Masashi Akiyama is an Editorial Board member of *Journal of Dermatology* and a co‐author of this article. To minimize bias, they were excluded from all editorial decision‐making related to the acceptance of this article for publication.

## ETHICS STATEMENT

Patient consent for publication was obtained. IRB approval status: Reviewed and approved by the ethics committees of Nagoya University Hospital and Toyohashi Municipal Hospital (approval numbers: 2022‐0422 and 736, respectively).

## Supporting information


Supporting Information Data S1.

## Data Availability

Data available on request.
