# Supplementary material for: Infiltration of M2a macrophages is predominant in genital verruciform xanthoma
Source: J Dermatol. 2025 Feb 6;52(3):556–8. doi: 10.1111/1346-8138.17654 (PMC11883849; doi:10.1111/1346-8138.17654)
Supplement: Supplementary file 1 — Supporting Information Data S1. [file JDE-52-556-s001.pdf]

Supplementary Figure 1. Clinical and pathological features of the skin lesions (Case 1).

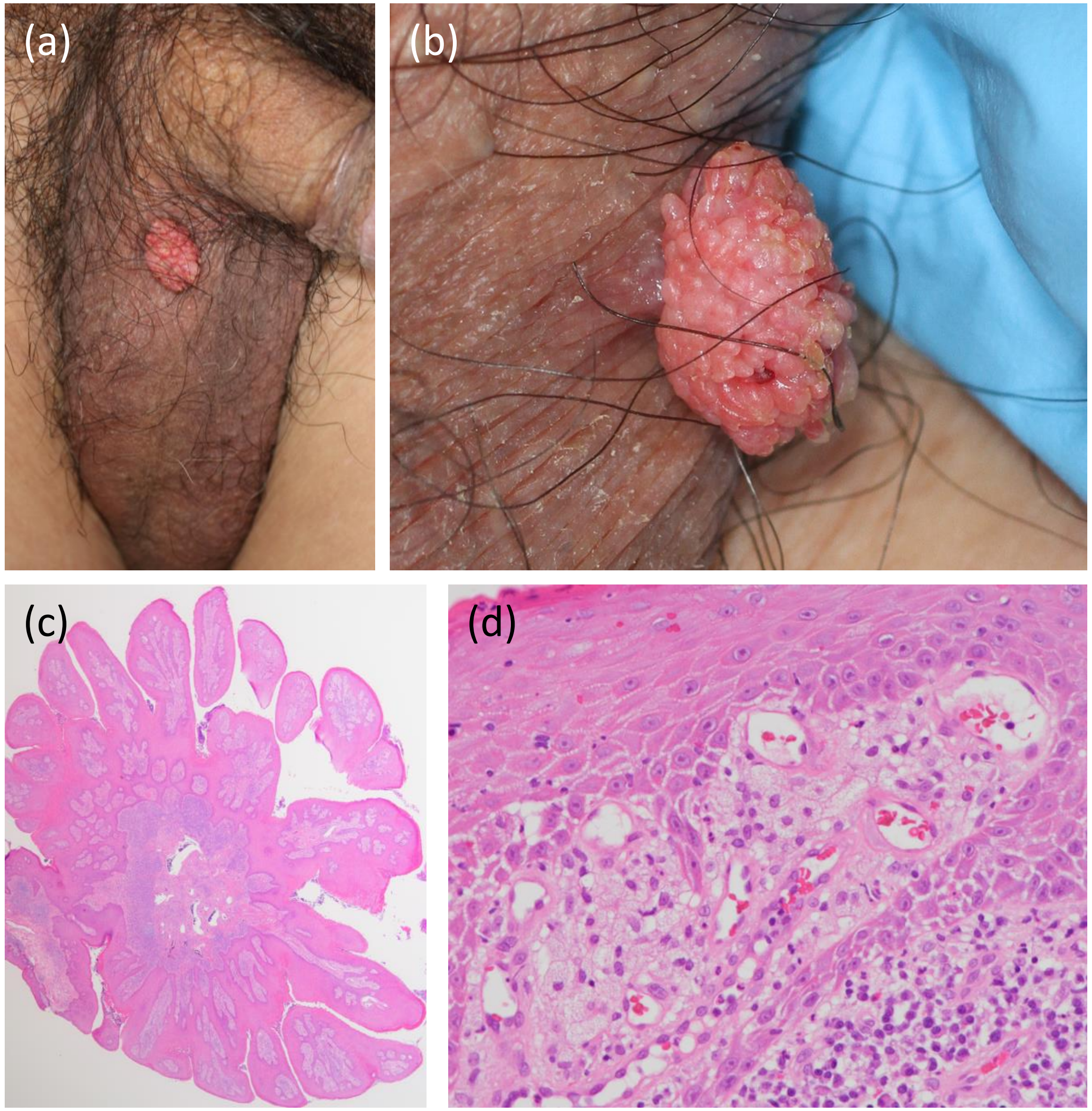

(a,b) A 1.5-cm × 1-cm erythematous pedunculated verrucous nodule on the scrotum.
(c,d) Hematoxylin–eosin (HE) staining shows abundant foamy macrophages in the dermis.

Supplementary Figure 2. Immunohistochemical features of the macrophage phenotypes (Case 1).

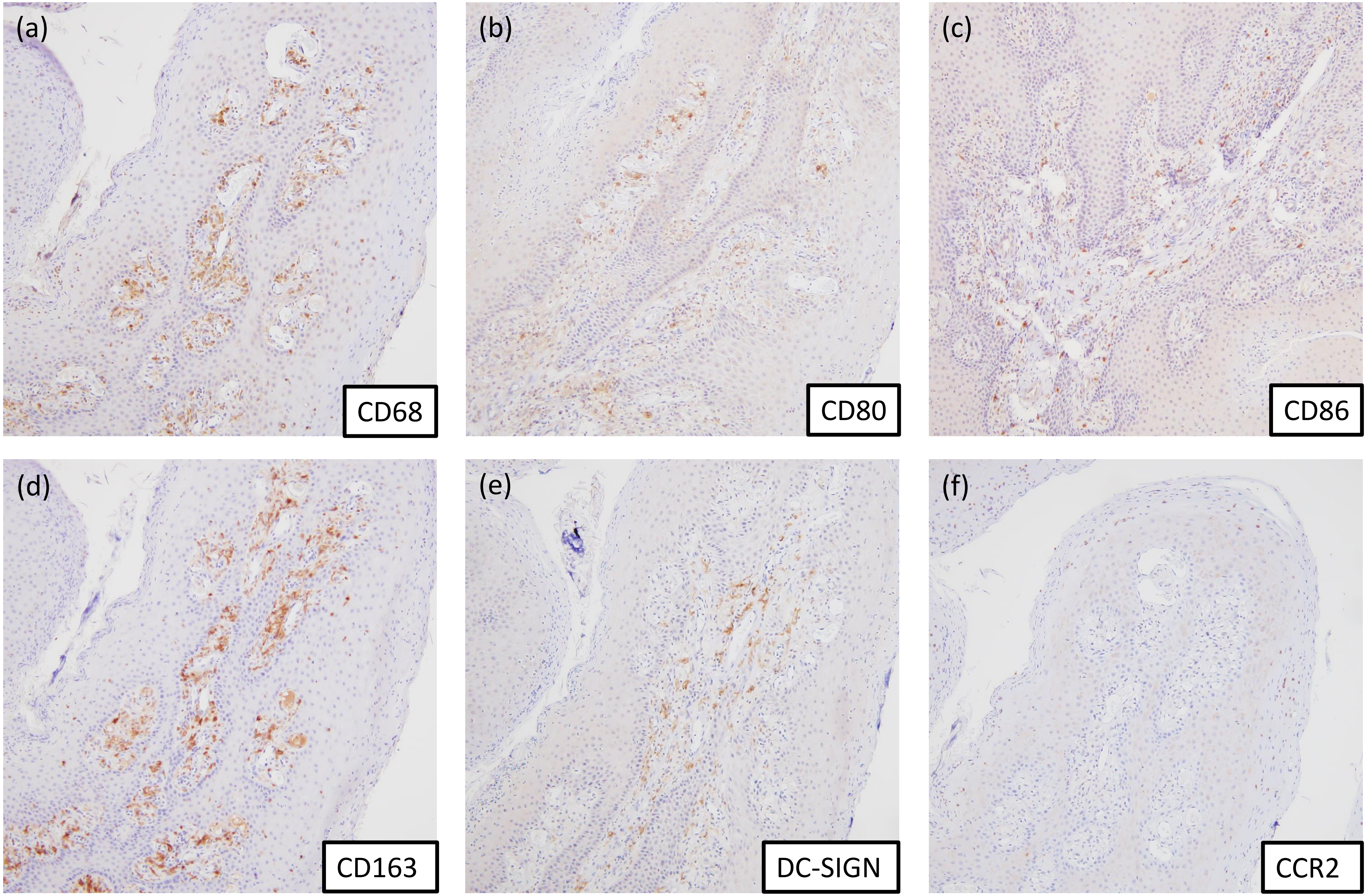

Macrophages are extremely positive for CD68 and CD163, strongly positive for DC-SIGN, positive for CD80 and CD86, and negative for CCR2.

Supplementary Figure 3. Immunofluorescence for the macrophage phenotypes and immunohistochemical features of type-2-related molecules (Case 1).

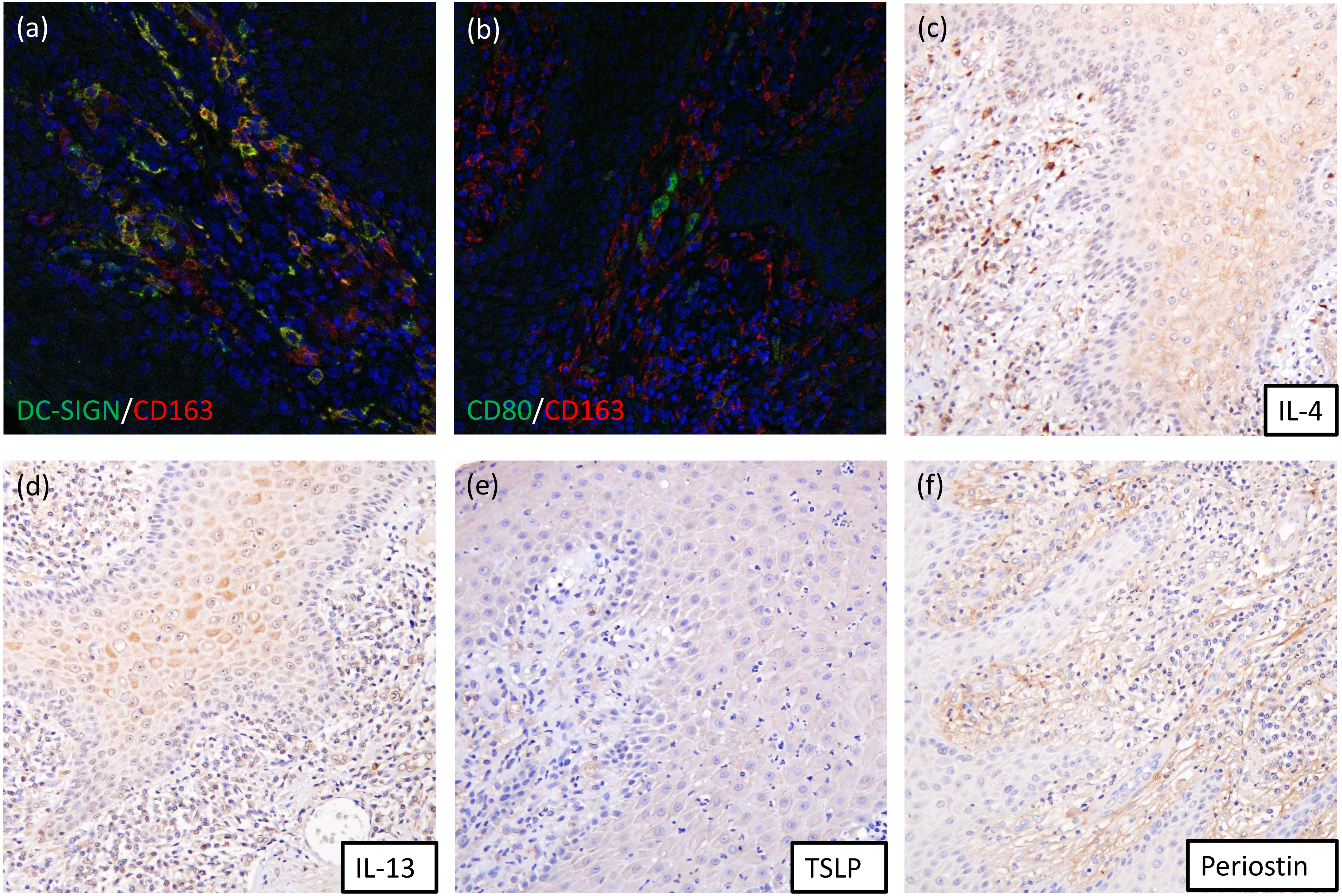

(a) A large proportion of DC-SIGN-positive cells are also positive for CD163 (yellow fluorescence indicating colocalization). (b) CD80-positive cells (green) and CD163-positive cells (red) exist independently. (c-f) Expression of type-2-related molecules.

Supplementary Figure 4. Immunohistochemical features of type-2-related molecules (healthy control).

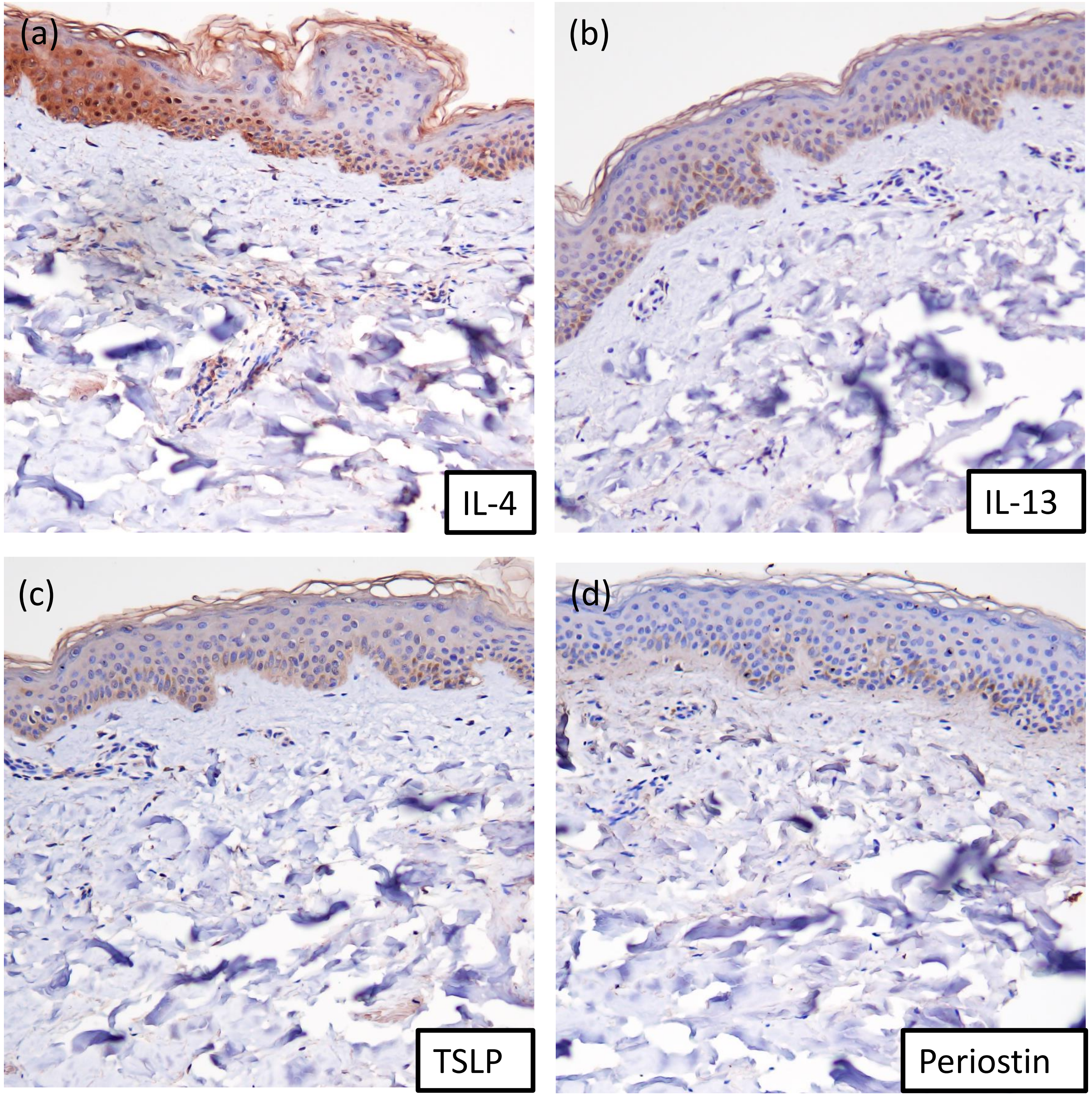

(a-d) Expression of type-2-related molecules.

Supplementary Figure 5. Quantification of type-2-related molecules in the verruciform xanthomas (case1-3) and the healthy control.

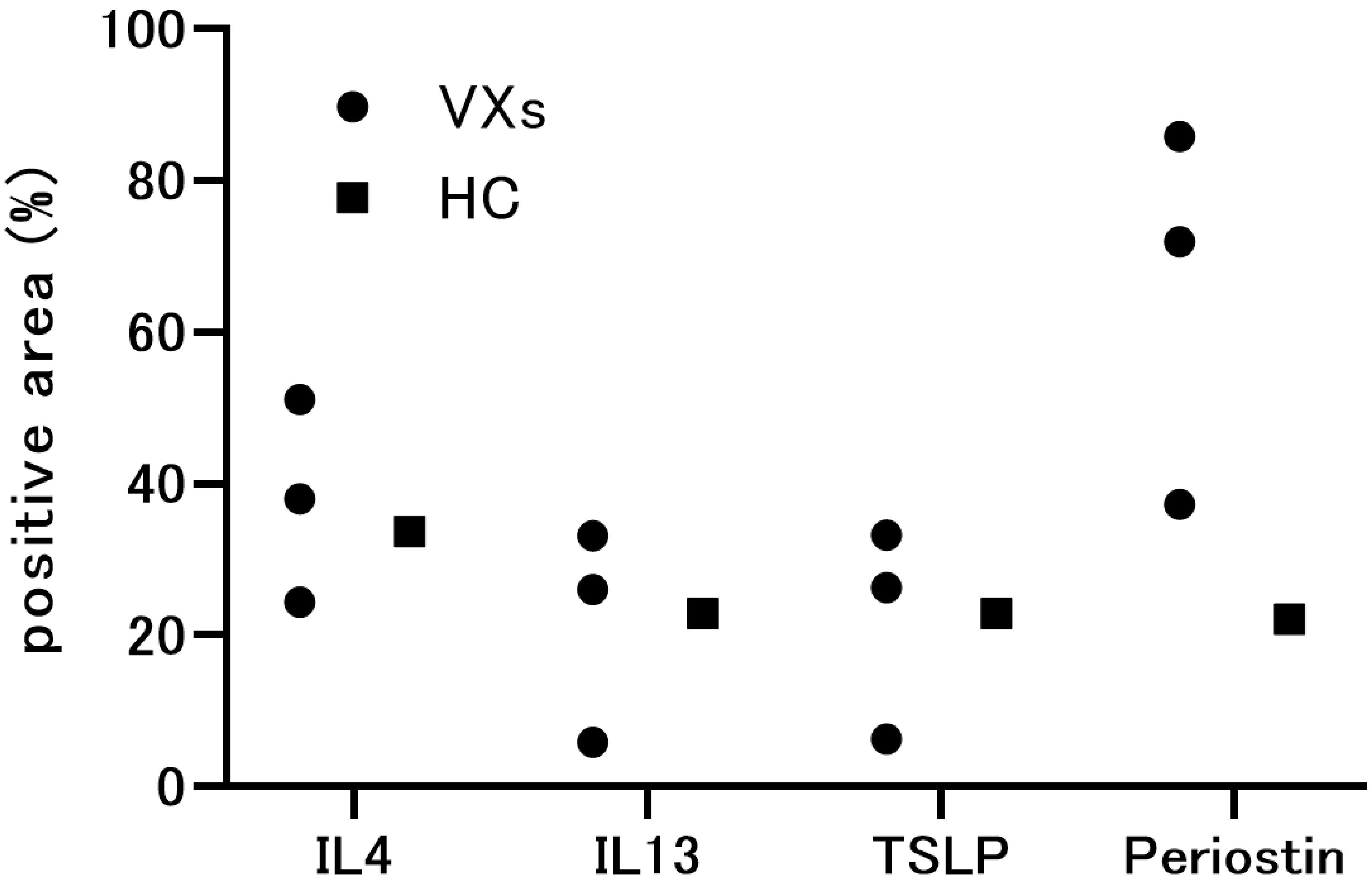

Immunohistochemically positive areas were quantified using HALO (Indica Labs, Albuquerque, NM). IL-4/13 and TSLP staining included the epidermis and the dermis, while periostin staining included only the dermis. Periostin was expressed more strongly in the patients than in the healthy control. VX, verruciform xanthoma; HC, healthy control.

Supplementary Figure 6. Pathological features of the skin lesions (Case 2).

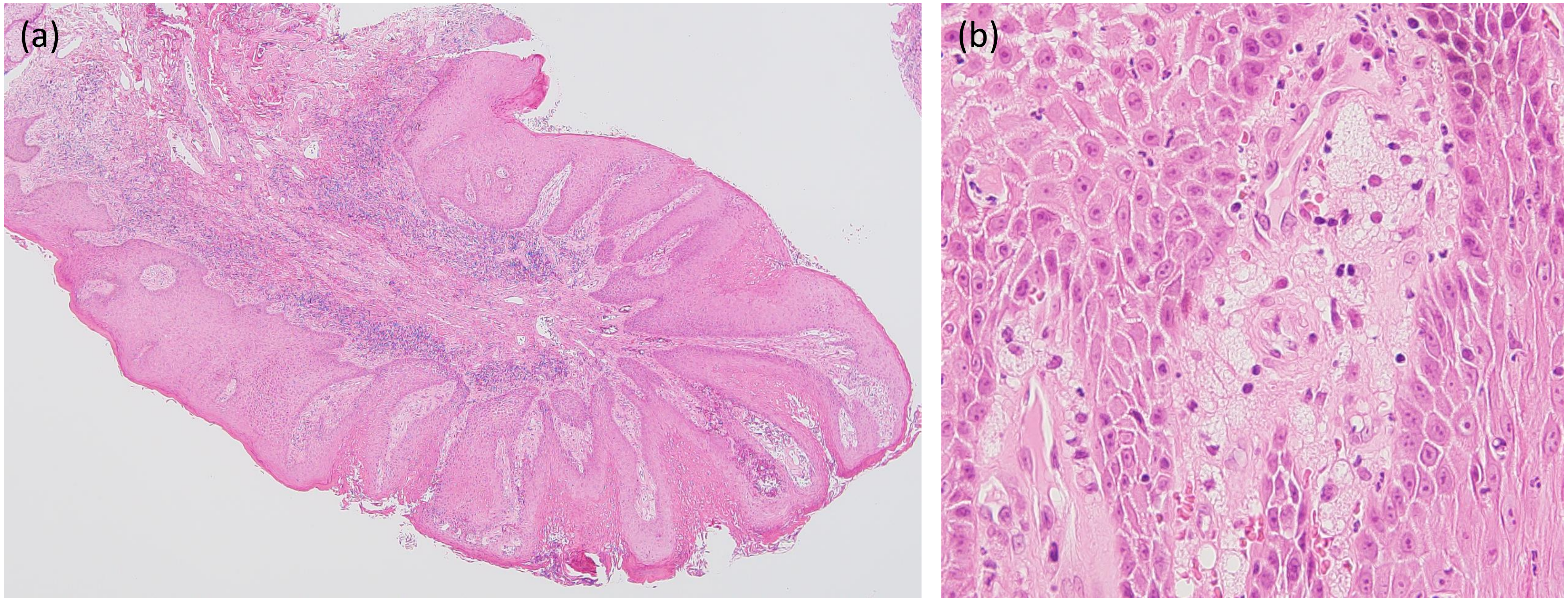

(a,b) HE staining shows marked papillomatosis of the epidermis and infiltration of foamy macrophages in the dermis.

Supplementary Figure 7. Immunohistochemical features of the macrophage phenotypes (Case 2).

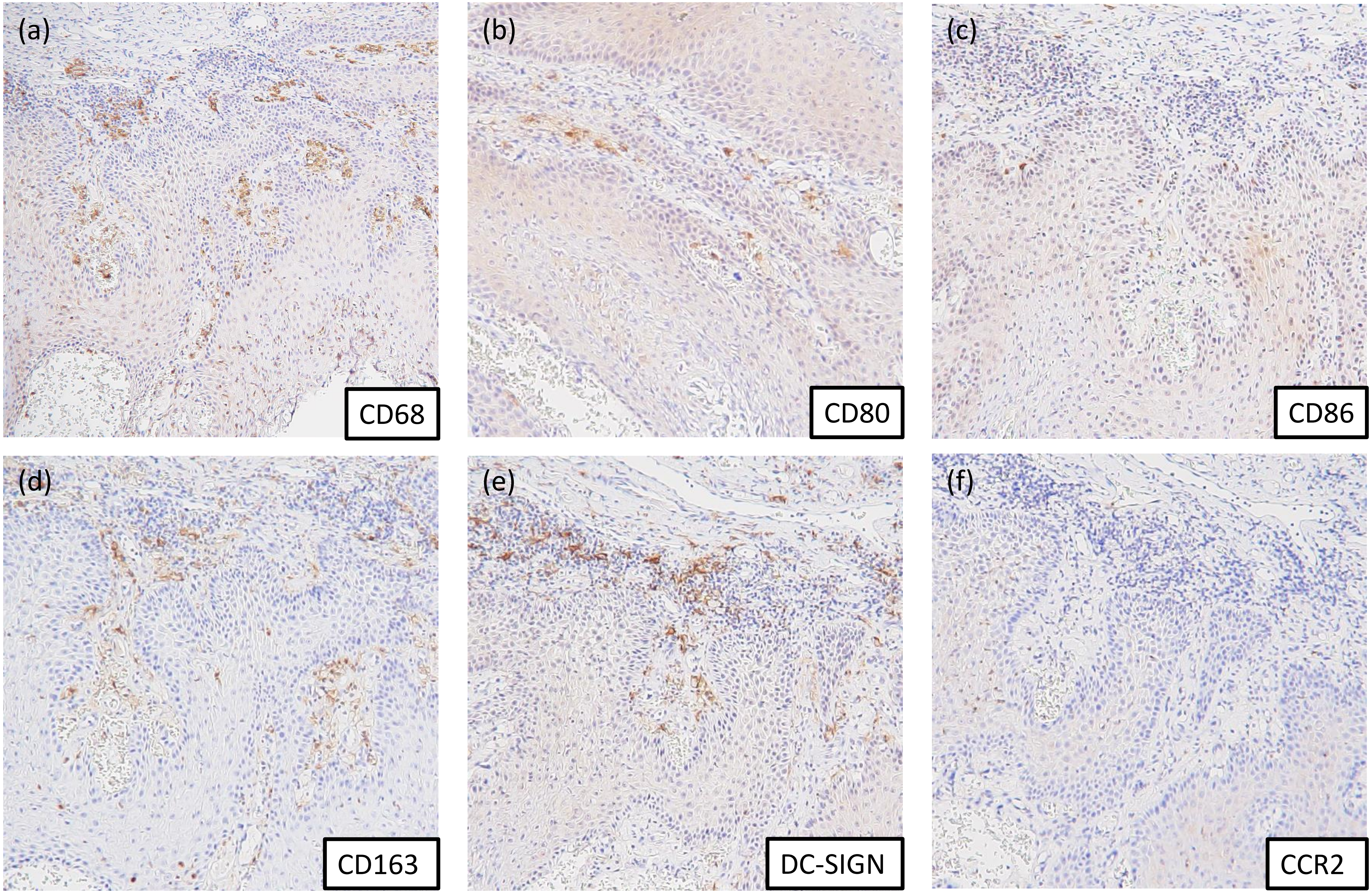

Macrophages are extremely positive for CD68, strongly positive for CD163 and DC-SIGN, positive for CD80 and CD86, and negative for CCR2.

Supplementary Figure 8. Immunofluorescence for the macrophage phenotypes and immunohistochemical features of type-2-related molecules (Case 2).

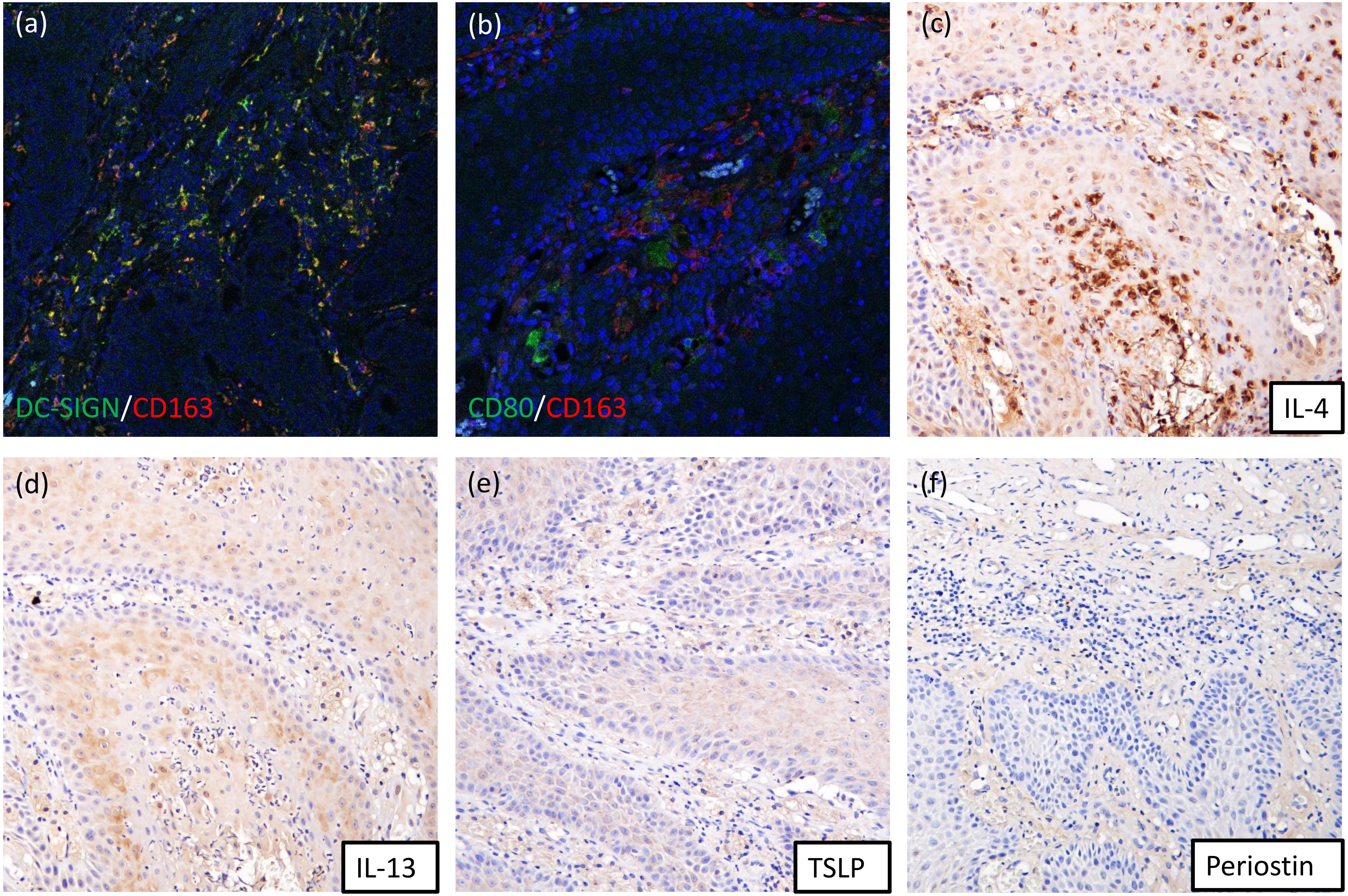

(a) A large proportion of DC-SIGN-positive cells are also positive for CD163 (yellow fluorescence indicating colocalization). (b) CD80-positive cells (green) and CD163-positive cells (red) exist independently. (c-f) Expression of type-2-related molecules.

Supplementary Figure 9. Clinical and pathological features of the skin lesions (Case 3).

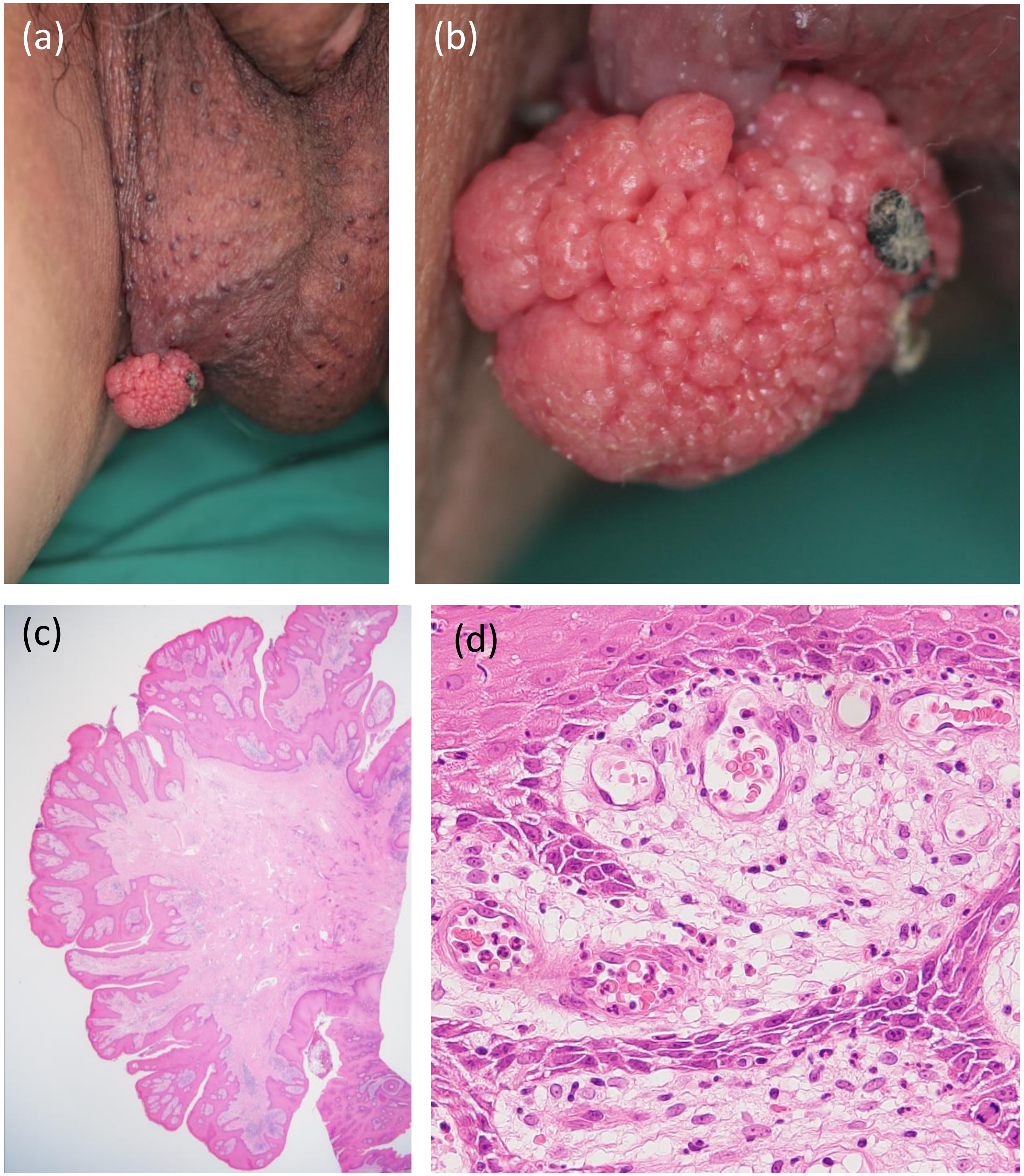

(a, b) A 2-cm erythematous pedunculated verrucous nodule. (c,d) HE staining shows papillomatosis of the epidermis and the infiltration of foamy macrophages in the dermis.

Supplementary Figure 10. Immunohistochemical features of the macrophage phenotypes (Case 3).

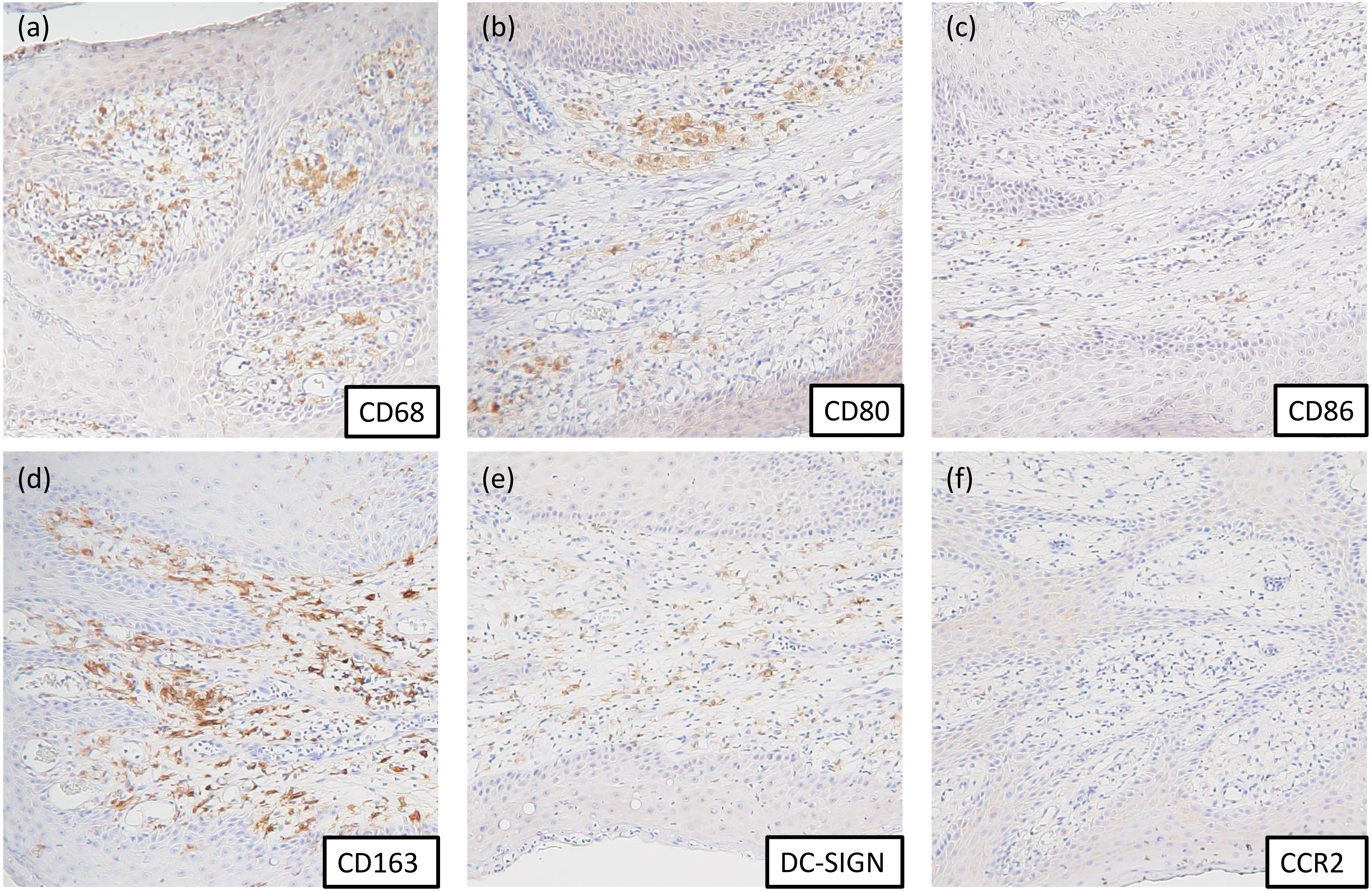

Macrophages are extremely positive for CD68 and CD163, strongly positive for CD80, positive for DC-SIGN and CD86, and negative for CCR2.

Supplementary Figure 11. Immunofluorescence for the macrophage phenotypes and immunohistochemical features of type-2-related molecules (Case 3).

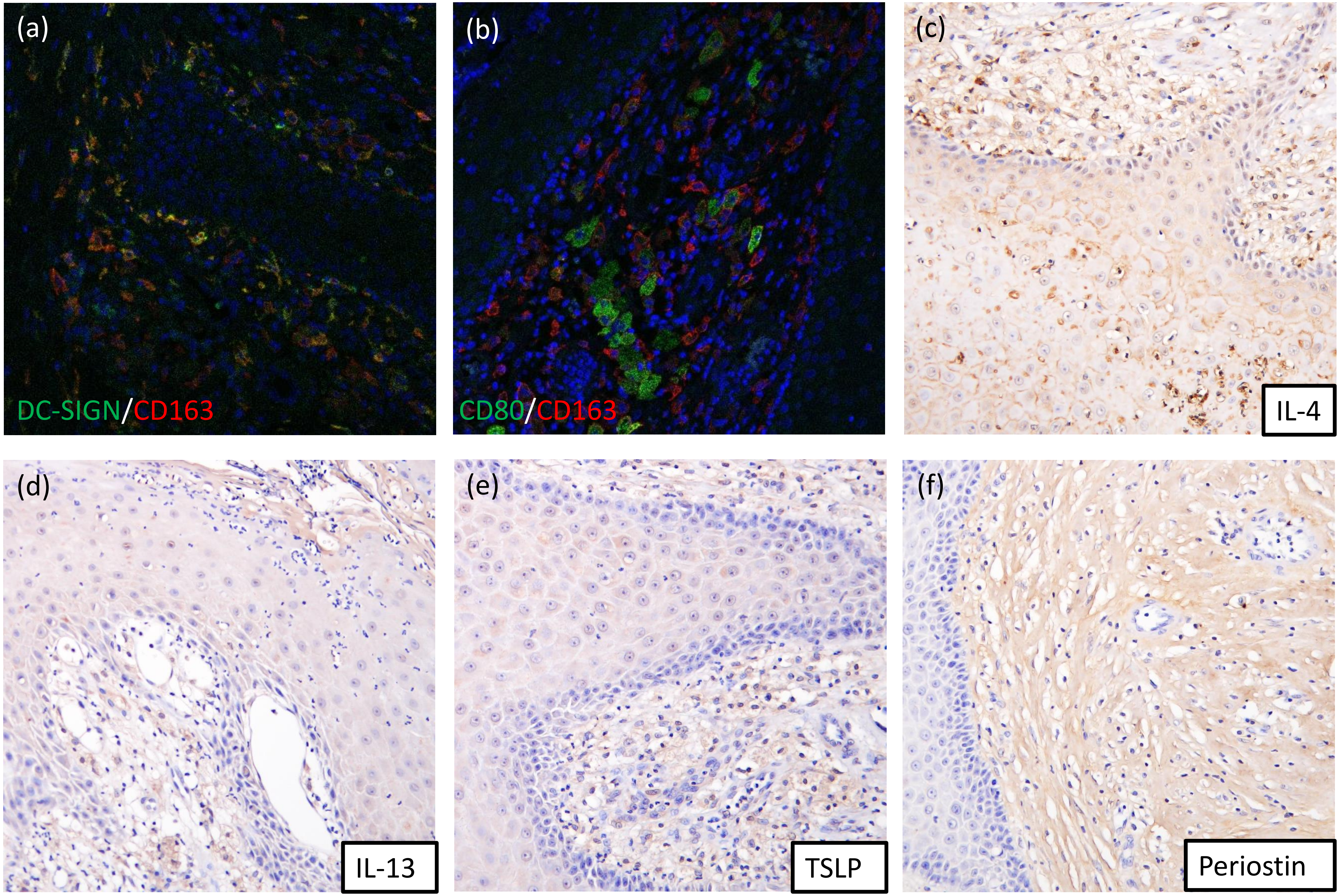

(a) Co-localization of DC-SIGN (green) and CD163 (red). (b) CD80-positive cells (green) and CD163-positive cells (red) exist independently. (c-f) Expression of type-2-related molecules.

Supplementary Figure 12. Immunohistochemical of the macrophage phenotypes (control, xanthelasma palpebrarum).

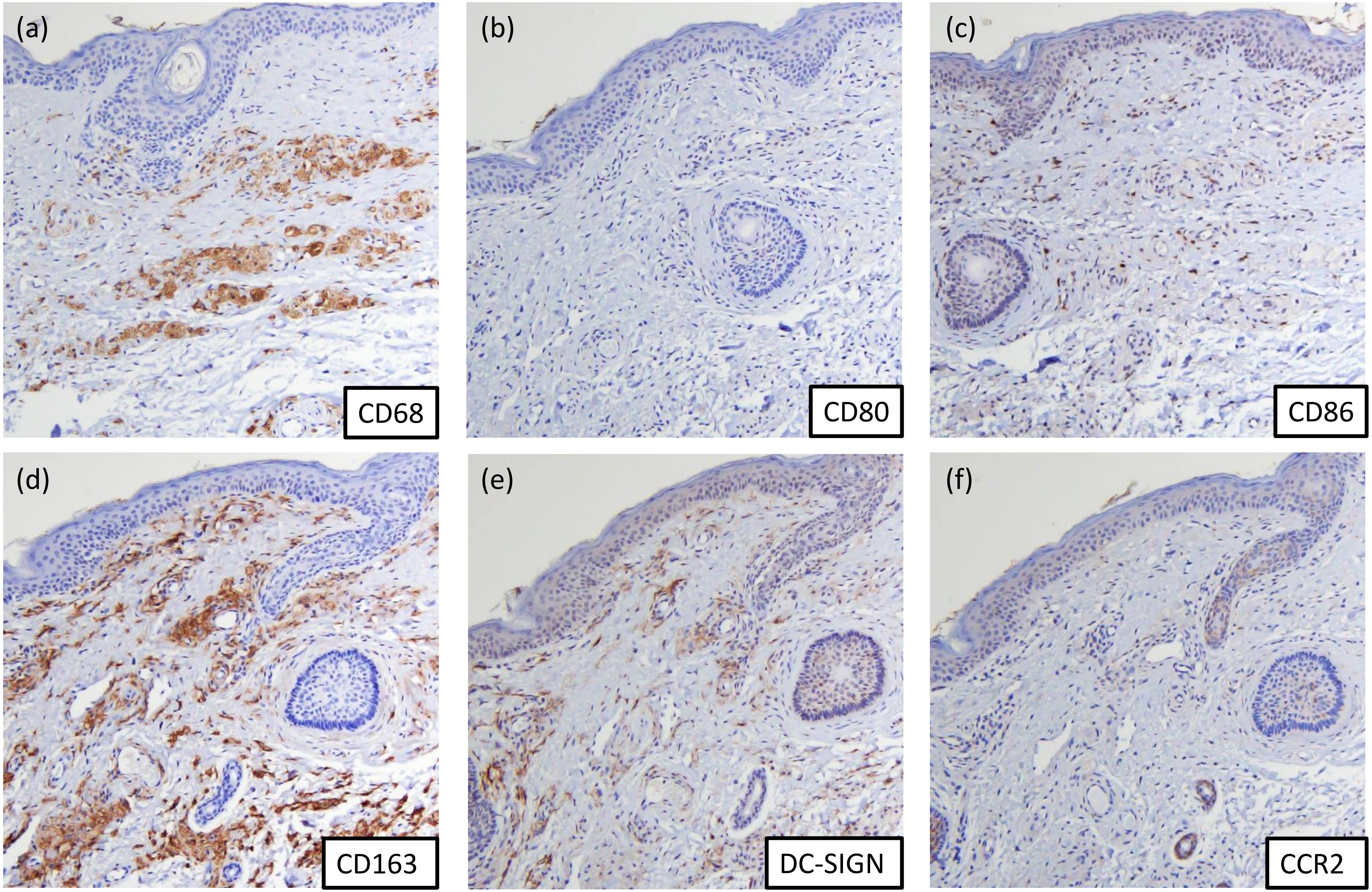

Macrophages are extremely positive for CD68 and CD163, positive for DC-SIGN and CD86, and negative for CD80 and CCR2.

Supplementary Figure 13. Immunohistochemical features of type-2-related molecules (control, xanthelasma palpebrarum).

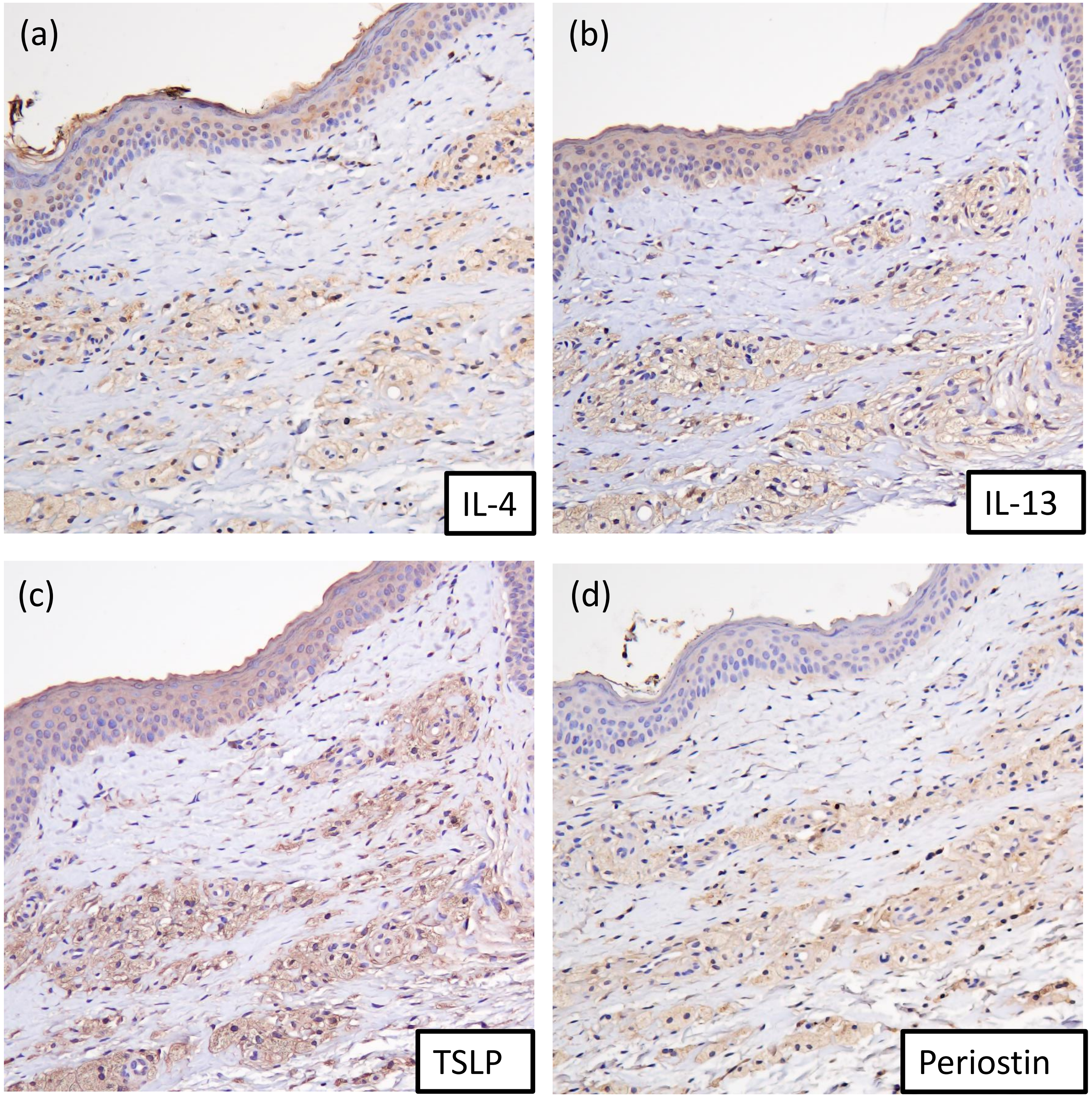

(a-d) Expression of type-2-related molecules.
